# The Truth Forcibly Spoken

**Published:** 1872-03

**Authors:** 


### THE TRUTH FORCIBLY SPOKEN.

At the last annual meeting of the Medical Society of the State of New York, which was held at Albany, N. Y., Feb. 6th ult,, Dr. Wm. C. Wey, the President, in his inaugural address, spoke as follows :

"The immediate and remote effects of medical fellowship are illustrated by the history of this society. Insensibly, but with great power, it has exercised its influence upon every section of the State. It has so materially elevated sentiment in remote districts, under the form of combination among medical men, even though few in number, that the greater and more enlarged interests of the profession have been conserved and perpetuated. An element like a medical organization, regularly and earnestly sustained, has so often proven the means of resisting error among the people, of inducing obedience to the standard of ethics and doctrine and of individual profit and advantage to its members, that its value cannot be adequately estimated.

"No one who has observed the evidence of medical growth and strength will question that incalculable good develops out of professional intercourse that has passed beyond the technicalities of constitutional amendments and the alteration of by-laws, and attained a condition that savors absolutely of legitimate scientific improvement. The fact has been repeatedly demonstrated, in a survey of the medical societies of the State, that the provisions of the code of ethics are most faithfully and conscientiously observed, and the personal standard of character ranks highest in those counties and organizations in which active, hearty fellowship is maintained. I may add respecting the code of ethics adopted by this society, and antedating all other American codes, which, in some quarters has been ridiculed and despised as effeminate and circumscribed in its teaching, that it is grounded upon a basis of morals which comprehends and illustrates the golden rule, and embraces in its scope an epitome of Christian teaching.

The importance and great value of medical organization upon the true basis, and for the purpose of erecting a high moral standard among its members, is fully demonstrated by the harmonious and practical workings of the State Society of New York.

We are gratified to know that the profession of that State are harmoniously united upon those principles, which alone, can cement its members into one great, efficient and aggressive brotherhood.

God forbid that any medical gentleman or organization should deem it necessary or legitimate to appeal to selfishness, or to the logic of the "almighty dollar," to induce our brethren to organize and maintain medical societies, and to obey and enforce the Code of Medical Ethics. It would seem to a mind enthused with admiration, in view of all the ennobling virtues of an exalted and Christian manhood, a work of supererogation, if not a degradation, to call up before the vision of such a soul, an argument so unworthy its dignity and character. We delight to take a loftier survey of the excellencies and glories of our profession: and yet were an appeal made alone to the most groveling of passions —that of self-interest—the facts to sustain the numberless advantages arising from a strict adhesion to honesty, and that sublime morality embodied in medical ethics, would be irresistably convincing! Look at the condition of the profession of New York, as depicted by one of its noblest members—Dr. Wey. Does not the wish well up from every truly professional heart, "would that our State enjoyed so glorious an heritage—one beautified and adorned by the medical virtues of its brotherhood—admired and respected by an appreciative constituency!"

Now, we desire to point out several reasons, which catch our eye in the address referred to, why the profession of New York enjoy such professional repose and popular respect:

1st, In vain does any master builder attempt to erect an edifice upon any other than the surest foundation; and any organization, whether religious, political, or medical, must, to defy destruction, rest upon a foundation of principle—not what men may choose to *style principle*—but upon those recognized truths which have emanated from the Great I Am. No organization can prosper long *void* of this *leaven*, and when this is cast aside for policy or expediency, its days are numbered and its doom certain. Hence the leaven of principle, utilized by a fraternal, united purpose for good to the profession and humanity, has "insensibly, but with great power," "exercised its influence upon every section" of New York, "by elevating sentiment" and "enlarging the interests of the same," which all see and feel have been "conserved and perpetuated" thereby.

2nd, A thorough medical organization, based upon the principles of the code, and blind, like the Goddess of Justice, in the interpretation and enforcement of its laws, "regularly and earnestly sustained," *does*, beyond question, "mobilize" the forces of the profession, which, when wisely handled, become the "means of resisting error among the people," and *does* exert a great moral influence by "inducing obedience to the standard of ethics," with the result—mark it!—"of individual profit and advantage to its members," the "value" of which "cannot be adequately estimated."

3d, That "a survey of the medical societies" of that State demonstrates "that the provisions of the code of ethics are most faithfully and conscientiously observed," and, as a legitimate result, "the *personal standard of character ranks high*, and hearty fellowship is maintained." And why? The veriest simpleton can answer: Because the State Society "is grounded upon a basis of morals which comprehends and illustrates the golden rule, and embraces in its scope an epitome of Christian teaching."

In view of the exalted status of the profession circumscribed by the State lines of New York, we, in common with all good men, must deplore the want of professional harmony and brotherhood in other sections. What the State Medical Society is to New York, so should the American Medical Association be to the Union. The question, therefore, naturally arises, Why does not the same unity of purpose and observance of the ethics prevail over the entire Union, under the workings of the National organization? We propose *here*, to give briefly, but one reason, out of many others, in solution of the question: and this consists in a want of devotion to the principles of the ethics which refuses to look beyond home to espouse the cause of those who are contending for them. Our brethren in many sections ignore the claims of their fellows, and deny them aid or sympathy, when battling for the very principles which they laud and endorse at home. Nay—more than this—they frequently extend co-operation and the influence of their names to elevate the enemies of the very principles they profess to admire, and by this means to crush, if possible, those who, with tension of "brain and muscle," are struggling to exalt the truth above the heads of those who spit upon and defy it. They, unwittingly, encourage rebellion in our ranks, and confer a quasi-respectability upon those who *dare* not defend them, without condemning themselves. With one hand these gentlemen, while holding aloft the ethics at home, with the other are innocently and unintentionally aiding in throttling it abroad. Under the protectorate of their good names an affiliation is claimed, the cause damaged, and the ethics trampled upon. Surely our brethren will pardon us for saying, their friends and the ethics deserve a better fate.

Dr. Wey remarks:

"To such an extent am I persuaded of the necessity of a more enlarged and specific knowledge and understanding of the ethics of our profession, that I would gladly see the whole subject made a branch of medical teaching in the schools, as a positive and necessary part of the means of education of the students."

Having so earnestly advocated the inestimable value of professional morality, we heartily endorse the recommendation to make the whole subject a branch of medical teaching. This will, doubtless, meet with opposition from certain quarters. An infidel scoffer in the Christian pulpit would be an anomaly. In all soberness and truth, such a man could not teach the Christian ethics, were he to attempt it; and it would be equally absurd to suppose that men who have for long years made it a study to deride and denounce medical ethics, could teach something so foreign to their feelings and so utterly opposed to their habits of thinking. It would be like Dan Rice running a tilt against the show business, or Barnum spouting a sermon against the evils of humbugery.

Dr. Wey continues:

According to the methods of instruction now practiced, inspiration of honor and character may be communicated to a pupil through the daily consistent walk and conversation of his preceptor. This ideal of excellence may be carried through the period of instruction and be incorporated in the professional life of the student, and it is a notable fact that if his former educational advantages have been liberal, his perception and appreciation of medical ethics will be immeasurably increased. On the other hand, if without previous preliminary preparation his early life has been devoted to practical rather than educational duties, and if it is his misfortune to pursue his studies under the training of a physician whose aim and purpose it is to procure business, regardless of the manner in which it is obtained, it is easy to see how, at the very beginning of his career, he is made to acquire false views of morality, honor and professional decorum, which subsequent lectures and introduction are in no way calculated to remove.

"In the first case the student, by reason of adequate education before taking up the study of medicine, is instructed in elements which, under proper direction, will produce the full development of professional character and learning, and, as a natural consequence, a jealous estimate of the ethics by which he is to be governed in his intercourse with his associates and patrons.

"In the other instance the whole subject of medical ethics remains a sealed book to the pupil, simply because his former life tended in no degree to prepare him for its comprehension. It was not illustrated, except by violation, and, as a matter of course, during his term of study, had received no explanation or illumination in the ordinary lecture term of a medical college.

"Considering the importance of this subject in comparison with many upon which much time is spent in medical colleges, its present and remote bearing on the pupil, on the profession at large, and on the public generally, I cannot but hope that it will ultimately receive the earnest attention of educators, so as to become an incorporated feature of teaching in the schools.

The vast importance of correct moral training of students upon the future of the profession cannot be estimated. We hail with pleasure, therefore, the noble and manly utterances of Dr. Wey upon this point. Last December we wrote as follows :

"The future of the profession, in a large measure, lies in the cultivation of the *moral* attributes of the students committed to the care of physicians by their parents. We should make them gentlemen as well as doctors, and in proportion as we succeed in accomplishing the first, so will we the other—and in elevating the dignity of the profession.

No parent, unless he desires his son to become a thief, would subject him to the pernicious example and maxims of the notorious swindlers of "Peter Funk" shops. Such a step would result in the inevitable ruin of the son, and be a calamity to the country and his friends. In medical training and education, example has much to do with the future of the medical student. If his preceptor is pure in thought and word—a man of unswerving integrity and truth, these great principles will be impressed upon him for life—and a gentleman and a scholar will be begotton under the hallowed influence of such a mind and heart.

But the moral training does not stop here Many instructors in our colleges, lose sight of the moral dignity they should impart as the highest stimulus to intellectual effort, in their teachings and general deportment. They should teach by example as well as by precept. The bright jewel of truth should sparkle and glow in their words and character; and the pure, spotless integrity of the Christian gentleman should pervade the lecture-room, no less than the private walks of life."

Dr. Wey says, further:

"A spirit has been manifested, where we would not have looked for evidence of such disregard of the obligations of professional obedience and discipline, to cut short the process of investigation of preferred charges against a member, in a properly qualified medical society, by an appeal to the civil courts to restrain the course of the inquiry by a summary injunction. Such a procedure is subversive of morals as well as justice, and effectually puts an end to all attempts to enforce compliance with the salutary rules by which voluntary and chartered organizations are sustained and perpetuated. It strikes at the very root of authority, by first assuming to be loyal and subject to it and afterward rebelling against its power in a way so treacherous and cowardly that but one course is left for a society thus compelled to yield to the interference of the officer of the law, and that is promptly to expel the revolutionary member; or if prevented by additional legal hindrances, to cease to hold social or professional relations with him."

For over twelve months this journal has had the painful, self-enforced duty of bearing testimony to the "disregard of the obligations of professional obedience and discipline" in many sections of the country—and, unfortunately, in Georgia. God forbid that, without cause, we should become "an accuser of our brethren!" To us the necessity of thus arraigning our erring brethren before the great tribunal of professional opinion has been the most painful duty of our professional life. Far more pleasant and gratifying would it have been, could we have uttered words of approval, and thus riveted the bonds of fraternal affection. Nothing but the vast interests at stake, and the heart-felt desire for the conservation and preservation of a profession whose escutcheon should bear no spot to mar its dignity and glory, could have drawn from us a record so damaging to its reputation and honor before an enlightened people. At best the medical profession holds no very sacred position in the popular heart. Charlantism preys upon the ignorance of thousands well informed in all else but medicine. Next to the Church, through whose instrumentality the world must be redeemed, medicine, through whose means the human body must be saved from death, is the Devil's mark of malignant hate. Scoffers at our science—like those at morals and religion—view with unfeigned delight the bickerings, wranglings and strivings among the *ministers* of mercy to suffering humanity!

Yet, to wage a warfare against evil is the Christian's lot. Even Jehovah bid the sun stand still, that by the day's lengthened hours the enemies of His cause might be punished.

And in what light shall the true men of the profession, in the unveiled presence of the truth, pouring its flood-tide of glory upon our ethics, view the assaults of diplomaed innovators upon the dignity and honor of so exalted a calling? Much the same as the pure men of the Christian Church would the apostacy of a brother to infidelity—and soul-ruin! In either case the cause demands fidelity on the part of the brotherhood, and an allignment on the side of truth, tempered with that "charity which *rejoices* in the truth." An honest effort should be made to induce the wanderer to repent, confess and abandon his error, and when such efforts fail, to then stigmatize his error, by "cutting him off."

In professional ostracism lies the only hope of the profession to either maintain its dignity or win the plaudits of an enlightened people. Less than this the cause of truth should not demand, more it might ask, which Dr. Wey *does* ask—even that of social ostracism.

